# Fulminant Pseudomembranous Colitis Leading to Clostridium Paraputrificum Bacteremia

**DOI:** 10.7759/cureus.13763

**Published:** 2021-03-08

**Authors:** Asim Haider, Fareeha Alavi, Ayesha Siddiqa, Hafsa Abbas, Harish Patel

**Affiliations:** 1 Internal Medicine, BronxCare Health System, Bronx, USA; 2 Gastroenterology, BronxCare Health System, Bronx, USA

**Keywords:** pseudomembranous colitis, clostridium paraputrificum bacteremia, fulminant colitis, clostridium difficle infection

## Abstract

*Clostridium* species are spore-forming gram-positive anaerobic rod bacteria that cause a broad range of infections in humans, including intra-abdominal infections, myonecrosis, and bacteremia. Pseudomembranous colitis (PMC) is a severe form of infection caused by *Clostridioides* *difficile*.* *Clostridial* *bacteremia usually occurs in the settings of neutropenia, alcohol abuse, diabetes mellitus, sickle cell anemia, malignancy, hemodialysis, inflammatory bowel disease, and AIDS. We report a case of fulminant PMC leading to *C. paraputrificum bacteremia *in an otherwise immunocompetent patient. To our knowledge, this is the first case report of such an occurrence*. *

## Introduction

Pseudomembranous colitis (PMC) is an inflammation of the bowel characterized by yellowish plaques on the intestinal mucosa leading to the formation of a pseudomembrane. It is usually but not always associated with *Clostridioides* (formerly *Clostridium*) *difficile* infection. Before the introduction of broad-spectrum antibiotics, PMC was associated with sepsis, colonic obstruction, cardiovascular insufficiency, heavy metal intoxications, shock, and uremia [1]. Although antibiotic-associated diarrhea may be common (10%-30%), PMC is far less frequent (1%-5%) [2]. *C. difficile* infection (CDI) symptoms include diarrhea, which is usually nonbloody, or colitis associated with severe abdominal pain, fever and/or gross or occult blood in the stools [2]. PMC, the severest form of this disease, occurs as a result of a severe inflammatory response to the *C. difficile* toxins [2].

*Clostridium* species are spore-forming gram-positive anaerobic rod bacteria that cause a broad range of infections in humans, including intra-abdominal infections, myonecrosis, and bacteremia. Clostridial bacteremia is often associated with underlying medical conditions, such as acquired immunodeficiency syndrome (AIDS), colonic malignancy, hemodialysis, and inflammatory bowel disease [3]. Among *Clostridium *species, *C. perfringens *(42%), *C. septicum* (14%), *C. ramosum* (9%), *C. clostridioforme* (6%), and *C. difficile* (5%) are the most common cause of bacteremia. In contrast, *C. paraputrificum* has been identified in only 1% of cases [3]. Takashi et al. in 2015 presented the first case of *C. paraputrificum* bacteremia associated with colitis in an immunocompromised patient with AIDS [4]. Herein, we present a case of *C. paraputrificum* bacteremia associated with PMC in an otherwise immunocompetent patient. 

## Case presentation

A 74-year-old male presented to the emergency department with the complaint of diarrhea for three weeks. The patient had non-bloody diarrhea (eight episodes per day) with no associated symptoms such as fever, abdominal pain, nausea, or vomiting. He had no history of sick contact, eating leftover food or recent travel. The patient had medical comorbidities of hypertension and iron deficiency anemia, for which he was on lisinopril and iron tablets. He was recently hospitalized for community-acquired pneumonia, for which he received seven days of ceftriaxone and five days of azithromycin. He denied any prior surgeries. He denied smoking, alcohol, or the use of recreational drugs. His review of systems was negative for prior history of diarrhea. The patient had a colonoscopy two years ago, which was unremarkable. On presentation, he was hypothermic with a temperature of 94.8 F, tachycardiac to 131/min, and hypotensive with a blood pressure of 80/38 mmHg. On physical examination, he was in mild distress. His cardiopulmonary, abdominal, and neurological exam were unremarkable. His Laboratory results were significant for leukocytosis (15 × 10^9^/L). The rest of the laboratory results were as following (Table 1).

**Table 1:** Pertinent laboratory findings. AST:  aspartate aminotransferase; ALT: alanine aminotransferase; HIV: human immunodeficiency virus; HBsAg: hepatitis B surface antigen; HBsAb: hepatitis B surface antibody; HCV: hepatitis C virus.

Lab parameter	Lab value	Reference range
White blood cells	15 × 10^9^/L	4-10 × 10^9^/L
Neutrophils	90%	40%-70%
Creatinine	1.6 mg/dl (baseline: 0.6)	0.5-1.5 mg/dl
Blood urea nitrogen	25 mg/dl	8-25 mg/dl
Serum albumin	1.7 g/dl	3.2-4.6 g/dl
Lipase	25 U/L	10 to 140 U/L
ALT	30 U/L	29 to 33 U/L
AST	33 U/L	5 to 40 U/L
ALP	80 U/L	44 to 147 U/L
HIV	Negative	Negative
HCV antibody	Negative	Negative
HBsAg	Negative	Negative
HBsAb	Positive	Negative
Total bilirubin	0.4 mg/dl	< 1.3 mg/dl
Serum sodium	137 mEq/L	135-145 mEq/L

**Figure 1:**
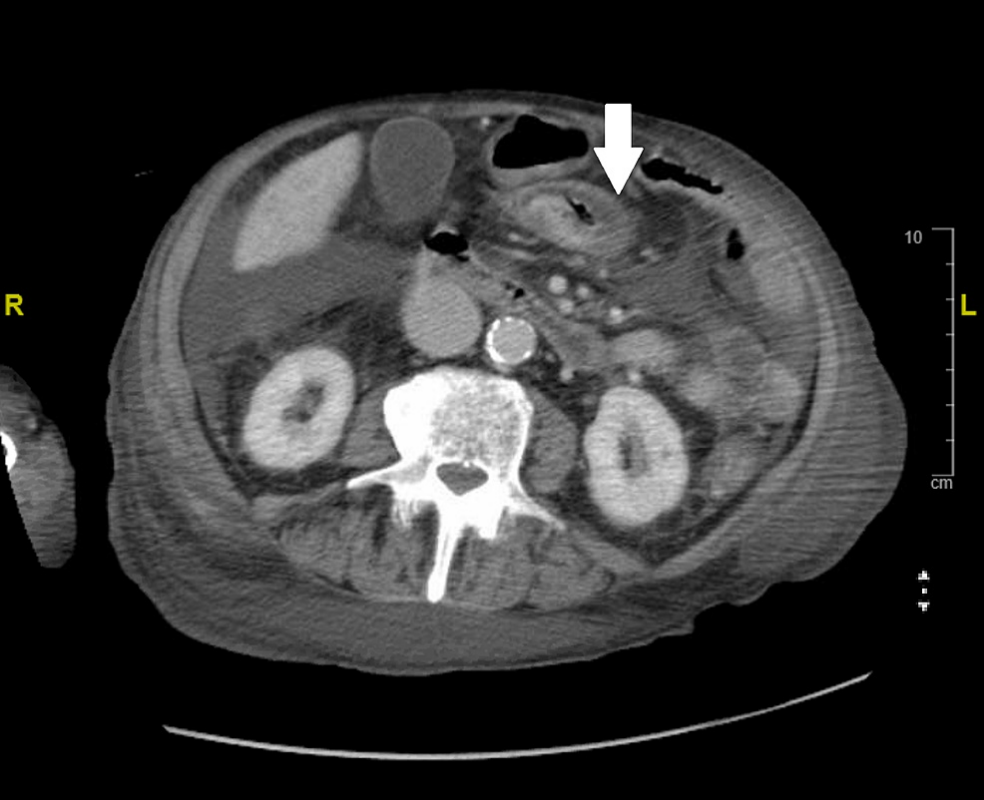
Computed tomography scan of the abdomen showing bowel wall thickening and surrounding inflammatory changes consistent with colitis.

A computed tomography (CT) scan of the abdomen and pelvis without contrast showed pancolitis. No Ileus or megacolon was noted in this imaging (Figure 1). 

**Figure 2:**
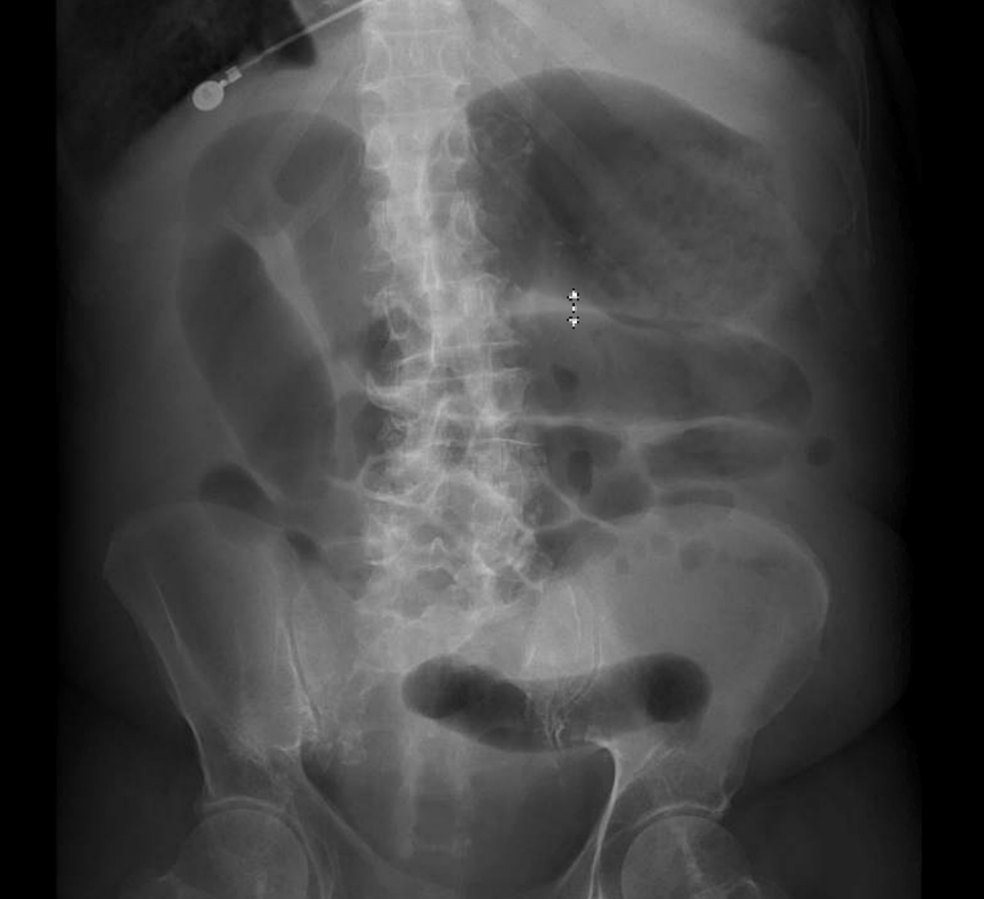
X-ray abdomen showing diffuse intestinal dilatation.

Septic workup (blood cultures and urine cultures) was sent, and the patient received 2L of the intravenous (IV) normal saline bolus. The patient did not respond to the fluid challenge and was eventually started on vasopressors. He was also started on the empiric treatment of fulminant CDI (oral vancomycin 500 mg every six hours and Intravenous metronidazole 500 mg every eight hours). The patient was admitted to the intensive critical unit. Patient's *C. difficile *toxin came back positive. The blood culture from admission grew *Clostridium paraputrificum.* Infectious disease was consulted, and oral vancomycin dose was decreased to 125 mg every six hours, and rectal vancomycin was stopped. On the 7th day of admission, the patient was still hypotensive, tachycardiac and his WBC count trended up to 20 x 10^9^/L. Over the day, the patient developed abdominal distention. X-ray abdomen showed diffuse dilatation of small and large bowel loops with concern for ileus. The intestinal dilatation was less than 6 cm and didn't meet the criteria for megacolon (Figure 2).

Rectal vancomycin was added to antibiotics regimen in view of ileus. Also, because of continuous septic shock requiring vasopressors, broad-spectrum antibiotics (vancomycin IV and meropenem IV) were started to cover any other possible source of infection. The nasogastric tube was placed for decompression. His condition eventually deteriorated, requiring mechanical ventilation. The palliative team was consulted per family wishes, and the patient was made do not resuscitate (DNR) and comfort measures. The patient eventually expired.

## Discussion

The gastrointestinal tract inhabits lots of bacteria [5]. Species of *Clostridium *represent the predominant bacteria in the gut and account for 10%-40% of the total microflora of gut [5]. They are the regulators of intestinal homeostasis. Most *Clostridium* species are commensal bacteria and live-in harmony with the intestinal environment. *Clostridium difficile* is the bacteria responsible for antibiotic-associated colitis. It usually colonizes the intestinal tract after transmission via the orofecal route. The colonization is facilitated by the disruption of the normal intestinal mucosa, mostly due to antimicrobial therapy [5]. *C. difficile* was identified as the causative bacteria in the majority of cases of antibiotic-associated colitis in 1978. From 1978 to 1983, the most common cause was clindamycin. Nonetheless from 1983 to 2003, the most commonly implicated antibiotics were cephalosporins [6]. There was a dramatic increase in frequency, severity as well as the rate of relapse of CDI from 2003 to 2006 [6]. Between 2011 and 2017, the incidence of healthcare-associated CDI decreased from 99.6 to 73.3 per 100,000 population [7]. The carrier rate of *C. difficile* is 3% among healthy adults, while it is as high as 8%-10% among hospitalized adults and long-term nursing home residents [8]. Asymptomatic *C. difficile *carriers are capable of shedding *C. difficile *spores and serve as a reservoir for environmental contamination to other hospitalized patients [9]. The transmission rate of *C difficile* is high, and it can be cultured readily from the hospital environment [10]. Patients harboring toxinogenic *C. difficile* (detected via nucleic acid amplification) in their stool are capable of serving as a source for transmission of infection to others, regardless of whether toxin is detected (via enzyme immunoassay) [11]. A study of *C. difficile *transmission using whole-genome sequencing has suggested that transmission is more likely to occur from patients with diarrhea related to active CDI than from patients with asymptomatic colonization [12]. 

The most important and easily modifiable risk factor responsible for CDI is antibiotics usage. Other established risk factors include advanced age, hospitalization, severe comorbid illness, enteral feeding, gastrointestinal surgery, obesity, cancer chemotherapy, hematopoietic stem cell transplantation, inflammatory bowel disease, cirrhosis, and possibly gastric acid suppression [6,13]. *C. difficile* diarrhea is mediated by genes for toxin A (tcdA) and toxin B (tcdB), which inactivate the Rho guanosine triphosphatases (Rho GTPases), leading to colonocyte death, loss of intestinal barrier function, and neutrophilic colitis. Serum antitoxin antibodies are the best-described host factor protecting against *C. difficile* pathogenesis. Asymptomatic carriers demonstrate higher serum levels of immunoglobulin IgG antibodies against toxin A than patients who develop clinical CDI. 

In contrast to *C. difficile*, *Clostridium paraputrificum* is an extremely rare species and constitutes only 1% of all *clostridium* infections in literature. *C. paraputrificum* is a part of normal intestinal flora, and mucosal barrier breakdown can result in clostridial bacteremia. Patients who develop *C. paraputrificum* bacteremia very often have underlying conditions that predispose them to infection, such as neutropenia, alcohol abuse, diabetes mellitus, sickle cell anemia, malignancy, and AIDS [14]. Clostridial bacteremia, more generally, is also frequently associated with underlying medical conditions such as colonic malignancy, AIDS, hemodialysis, and inflammatory bowel disease [3]. Although this patient had no apparent immunocompromised state, the presence of severe CDI was probably the source of *Clostridium paraputrificum* bacteremia. Increased colonic permeability due to severe colitis might be an important component of gastrointestinal dysfunction.

Due to the rarity of* C. paraputrificum *bacteremias rare, the general consensus treatment guidelines are not clear. Leal et al. studied the results of susceptibility testing of 135 patients with *Clostridium* species bacteremia during 2000-2006. Results showed that reduced susceptibility to metronidazole occurred in 2/135 (1%), to penicillin in 14/135 (10%), and to clindamycin in 36/135 (27%) isolates [3]. This data suggest that metronidazole should be added in all the patients with *Clostridium* bacteremia, and clindamycin should be avoided as an empirical treatment. Finally, the prognosis of *C. paraputrificum* bacteremias is unclear, again due to the rarity of the condition [14]. 

## Conclusions

Clostridial bacteremia usually occurs in the settings of neutropenia, alcohol abuse, diabetes mellitus, sickle cell anemia, malignancy, hemodialysis, inflammatory bowel disease, and AIDS. *Clostridium paraputrificum* is a part of normal intestinal flora. Damage to the intestinal mucous membrane by severe colitis can lead to *C. paraputrificum* bacteremia. We presented a case of fulminant PMC leading to *C. paraputrificum* bacteremia in an otherwise immunocompetent patient.
